# miRNA profiling of primate cervicovaginal lavage and extracellular vesicles reveals miR‐186‐5p as a potential antiretroviral factor in macrophages

**DOI:** 10.1002/2211-5463.12952

**Published:** 2020-09-11

**Authors:** Zezhou Zhao, Dillon C. Muth, Kathleen Mulka, Zhaohao Liao, Bonita H. Powell, Grace V. Hancock, Kelly A. Metcalf Pate, Kenneth W. Witwer

**Affiliations:** ^1^ Department of Molecular and Comparative Pathobiology The Johns Hopkins University School of Medicine Baltimore MD USA; ^2^ Department of Neurology The Johns Hopkins University School of Medicine Baltimore MD USA; ^3^ University of California Los Angeles CA USA

**Keywords:** cervicovaginal lavage, exosome, extracellular vesicle, HIV‐1, microRNA

## Abstract

Cervicovaginal secretions, or their components collected, are referred to as cervicovaginal lavage (CVL). CVL constituents have utility as biomarkers and play protective roles in wound healing and against HIV‐1 infection. However, several components of cervicovaginal fluids are less well understood, such as extracellular RNAs and their carriers, for example, extracellular vesicles (EVs). EVs comprise a wide array of double‐leaflet membrane extracellular particles and range in diameter from 30 nm to over one micron. The aim of this study was to determine whether differentially regulated CVL microRNAs (miRNAs) might influence retrovirus replication. To this end, we characterized EVs and miRNAs of primate CVL during the menstrual cycle and simian immunodeficiency virus (SIV) infection of macaques. EVs were enriched by stepped ultracentrifugation, and miRNA profiles were assessed with a medium‐throughput stem‐loop/hydrolysis probe qPCR platform. Whereas hormone cycling was abnormal in infected subjects, EV concentration correlated with progesterone concentration in uninfected subjects. miRNAs were present predominantly in the EV‐depleted CVL supernatant. Only a small number of CVL miRNAs changed during the menstrual cycle or SIV infection, for example, miR‐186‐5p, which was depleted in retroviral infection. This miRNA inhibited HIV replication in infected macrophages *in vitro*. *In silico* target prediction and pathway enrichment analyses shed light on the probable functions of miR‐186‐5p in hindering HIV infections via immunoregulation, T‐cell regulation, disruption of viral pathways, etc. These results provide further evidence for the potential of EVs and small RNAs as biomarkers or effectors of disease processes in the reproductive tract.

## Graphical abstract

By comparing the microRNA and extracellular vesicle profiles of cervicovaginal lavage of both uninfected and infected macaques, we observed an enrichment of microRNAs in extracellular vesicle‐depleted supernatant and discovered a significant reduction in miR‐186‐5p after simian immunodeficiency virus infection. Furthermore, once transfected into HIV‐infected human macrophages, synthetic miR‐186‐5p mimics inhibited HIV replication, demonstrating its antiretroviral potential.

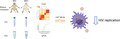

**Abbreviations**
ACD: acid citrate dextrose
CVL: cervicovaginal lavage
EV: extracellular vesicle
exRNAs: extracellular RNAs
exRNPs: extracellular ribonucleoprotein complexes
IFN‐γ: interferon‐γ
M‐CSF: macrophage colony‐stimulating factor
MDM10: macrophage differentiation medium with 10% FBS
MDM20: macrophage differentiation medium with 20% FBS
miRNAs: microRNAs
NTA: nanoparticle tracking analysis
PBMCs: peripheral blood mononuclear cells
RBC: red blood cell
SIV: simian immunodeficiency virus
TLDA: TaqMan low‐density array
UC: ultracentrifuge

The cervicovaginal canal is a potential source of biological markers for forensics investigations [1, 2, 3, 4], reproductive tract cancers [5, 6, 7], and infections [8, 9, 10]. Cervicovaginal secretions may be collected by swab, tampon, or other methods, or secretion components may be liberated by a buffered wash solution and collected as cervicovaginal lavage (CVL). In addition to utility as biomarkers, constituents of cervicovaginal secretions, including proteins, certain microbes, and metabolites, exert function, for example, by playing protective roles in wound healing [11] and against HIV‐1 infection [12, 13, 14, 15, 16, 17, 18, 19, 20, 21, 22]. A large and important body of work has thus examined biomarker potential and functional roles of numerous entities in the cervicovaginal compartment.

Compared with secreted proteins, metabolites, and the microbiome, however, several components of cervicovaginal fluids are less well understood, including extracellular RNAs (exRNAs) and their carriers, such as extracellular vesicles (EVs) and extracellular ribonucleoprotein complexes (exRNPs). EVs are potential regulators of cell behavior in paracrine and endocrine fashion due to their reported abilities to transfer proteins, nucleic acids, sugars, and lipids between cells [23]. EVs comprise a wide array of double‐leaflet membrane extracellular particles, including those of endosomal and cell‐surface origin [24, 25], and range in diameter from 30 nm to well over one micron (large oncosomes) [26]. EV macromolecular composition tends to reflect, but is not necessarily identical to, that of the cell of origin [27]. EVs have been isolated from most cells, as well as biological fluids [23, 28], including cervicovaginal secretions of humans [29] and rhesus macaques [30].

MicroRNAs (miRNAs) are one of the most studied classes of exRNA. These noncoding RNAs average 22 nucleotides in length and, in some cases, fine‐tune the expression of target transcripts [31, 32]. Released from cells by several routes, miRNAs are among the most frequently examined biomarker candidates in biofluids and, along with some other RNAs, are reported to be transmitted via EVs [33, 34, 35, 36]. miRNAs are found not only in EVs, but also in free Argonaute‐containing protein complexes; the latter may outnumber the former, at least in blood [37, 38]. Many miRNAs are also highly conserved [32], and abundant species typically have 100% identity in humans and nonhuman primates [39]. [For this reason, we will refer to hsa‐ (*Homo sapiens*) and mml‐ (*Macaca mulatta*) miRNAs without the species designation unless otherwise warranted by sequence disparity.] While miRNAs have been profiled in cervicovaginal secretions and menstrual blood, mostly in the forensics setting [4, 40, 41], their associations with EV and exRNP fractions require further study. A recent publication reported that EVs from healthy vaginal secretions inhibited HIV‐1 infection [29]. Another report found that CVL EVs (styled “exosomes”) were present at higher concentrations in cervical cancer and that two miRNAs were also upregulated [5]. Our laboratory described a reduction of CVL EVs in a severe endometriosis case compared with reproductively healthy primates [30]. However, our study, along with others, was limited by the absence of molecular profiling of EV cargo [30].

Many immunocompetent cell types populate the female reproductive tracts, among which are vaginal‐resident macrophages. These cells reside in the lamina propria and participate in the host's innate immune responses via specialized phagocytic elimination and limited antigen‐presenting capability [42, 43]. Once activated, for example, by sensing interferon‐γ (IFN‐γ) from Th1 effectors cells [43], vaginal macrophages increase their degradative ability. The recruitment of vaginal macrophages is sex hormone‐dependent, and their phagocytotic capability is not impaired by the low pH of the microenvironment [43, 44]. Numerous papers have demonstrated that CD68^+^ macrophages express receptors CD4, CCR5, and CXCR4, indicating that they are susceptible to infection by both R5 and X4‐tropic HIV‐1 virus during genital infection and transmission [42, 43, 45, 46, 47].

Here, we performed targeted miRNA profiling of EV‐enriched and EV‐depleted fractions of CVL and vaginal secretions collected from healthy and retrovirus‐infected rhesus macaques. We queried how CVL EVs and miRNAs are affected by the menstrual cycle, an important potential confounder of biomarker studies [48]. Similarly, we assessed possible associations with simian immunodeficiency virus (SIV) infection. We report an association of miR‐186 levels with SIV infection and find that this miRNA also appears to have antiretroviral effects in HIV‐infected macrophages. These studies provide baseline information for easily accessed CVL markers including EVs and miRNAs that may become useful tools in the clinic.

## Materials and methods

### Sample collection

Cervicovaginal lavage and whole blood samples were collected weekly for 5 weeks from two uninfected (control) and four SIVmac251‐infected (infected) rhesus macaques (*M. mulatta*) as previously described [30]. All macaques were negative for simian T‐cell leukemia virus and simian type D retrovirus and were inoculated intravenously. Animals were sedated with ketamine at a dose of 7–10 mg·kg^−1^ prior to all procedures. CVL was performed by washing the cervicovaginal cavity with 3 mL of PBS (Thermo Fisher Scientific, Waltham, MA, USA. Cat #: 14190‐144) directed into the cervicovaginal canal and re‐aspirated using the same syringe. Materials and procedures for sample collection are depicted in Fig. S1. Volumes of CVL yield across collection dates were documented in Table S1. Whole blood (3 mL) was collected by venipuncture into syringes containing acid citrate dextrose (ACD) solution (Sigma‐Aldrich, St. Louis, MO, USA. Cat #: C3821).

### Study approvals

All animal studies were approved by the Johns Hopkins University Institutional Animal Care and Use Committee and conducted in accordance with the Weatherall Report, the Guide for the Care and Use of Laboratory Animals, and the USDA Animal Welfare Act.

### Sample processing

Sample processing began within a maximum of 60 min of collection and utilized serial centrifugation steps to enrich EVs as described previously [30], based on a standard EV isolation protocol [49]. Specifically, fluids were centrifuged: (a) 1000 ***g*** for 15 min at 4 °C in a tabletop centrifuge; (b) 10 000 ***g*** for 20 min at 4 °C; and (c) 110 000 ***g*** for 2 h at 4 °C with a Sorvall Discovery SE ultracentrifuge (Thermo Fisher Scientific) with an AH‐650 rotor (*k* factor: 53.0; Fig. S1B). Following each centrifugation step, most supernatant was removed, taking care not to disturb the pellet. After each step, supernatant was set aside for nanoparticle tracking analysis (NTA; 200 µL) and RNA isolation (200 µL) following the second and third steps. The pellet was resuspended in 400 µL of PBS after each centrifugation step. After the final step, the remaining ultracentrifuged (UC) supernatant was concentrated to approximately 220 µL using Amicon Ultra‐2 10‐kDa molecular weight cutoff filters (Merck KGaA, Darmstadt, Germany. Cat #: UFC201024). Two hundred microliter of the concentrate was used for RNA isolation, and the remainder was retained for NTA. All samples reserved for RNA isolation were mixed with 62.6 µL of RNA isolation buffer (Exiqon, Vedbaek, Denmark. Cat #: 300112. Lot #: 593‐84‐9n) containing three micrograms of glycogen and 5 pg of synthetic cel‐miR‐39 as previously described [50]. Processed samples were analyzed immediately or frozen at −80 °C until further use.

For plasma, whole blood was centrifuged at 800 ***g*** for 10 min at 25 °C. Supernatant was centrifuged twice at 2500 ***g*** for 10 min at 25 °C. The resulting platelet‐poor plasma was aliquoted and frozen at −80 °C.

### Hormone analysis

Levels of progesterone (P4) and estradiol‐17b (E2) were measured in plasma samples shipped overnight on dry ice to the Endocrine Technology and Support Core Lab at the Oregon National Primate Research Center, Oregon Health and Science University.

### Nanoparticle tracking analysis

Extracellular particle concentration was determined using a NanoSight NS500 NTA system (Malvern, Worcestershire, UK). CVL samples were diluted as needed and specified in Table S2 to ensure optimal NTA analysis. At least five 20‐s videos were recorded for each sample at a camera setting of 12. Data were analyzed at a detection threshold of two using nanosight software version 3.0.

### Western blot

Western blot was used to detect the presence of EV protein markers and the absence of nucleoporin (nuclear marker) in CVL and enriched CVL EVs. Twenty microliter of samples from each fraction was lysed with 5 µL 1 : 1 mixture of RIPA buffer (Cell Signaling Technology, Danvers, MA, USA. Cat #: 9806S) and protease inhibitor (Santa Cruz Biotechnology, Dallas, TX, USA. Cat #: sc29131). Eight microliter of Laemmli 4× sample buffer (Bio‐Rad, Hercules, CA, USA. Cat #:161‐0747 Lot #: 64077737) was added per sample, and 30 µL of each was loaded into a Criterion TGX 4–15% gel (Bio‐Rad. Cat #: 5678084 Lot #: 64301319) after 5 min of 95 °C incubation. The gel was electrophoresed by application of 100 V for 100 min. The proteins were then transferred to a PVDF membrane (Bio‐Rad. Cat #: 1620177, Lot #:31689A12.), which was blocked with 5% milk (Bio‐Rad. Cat #: 1706404. Lot #: 64047053) in PBS + 0.1%Tween®20 (Sigma‐Aldrich, Cat #: 274348 Lot #: MKBF5463V) for 1 h. The membrane was subsequently incubated with mouse anti‐human CD63 (BD Biosciences, San Jose, CA, USA, Cat #: 556019 Lot #: 6355939) and mouse monoclonal IgG_2b CD81 (Santa Cruz Biotechnology, Cat #: 166029 Lot #: L1015) primary antibodies, at a concentration of 0.5 µg·mL^−1^ overnight. After washing the membrane, it was incubated with a goat anti‐mouse IgG‐HRP secondary antibody (Santa Cruz Biotechnology, Cat #: sc‐2005 Lot #: B1616) at a 1 : 5000 dilution for 1 h. The membrane was then incubated with a 1 : 1 mixture of SuperSignal West Pico Stable Peroxide solution and Luminol Enhancer solution (Thermo Scientific, Rockford, IL, USA, Cat #: 34080 Lot #: SD246944) for 5 min. The membrane was visualized on Azure 600 imaging system (Azure Biosystems, Dublin, CA, USA). The second blot was done in a reducing environment using 10 mm DTT (Promega, Madison, WI, USA, Cat #: P1171 Lot #: 0000198991). Same procedures were followed with rabbit anti‐human TSG101 (Cat #: ab125011 Lot #:GR180132‐14), rabbit polyclonal antinucleoporin (Abcam, Cambridge, MA, USA, Cat #: ab96134 Lot #: GR22167‐18) primary antibodies. Subsequent incubation with goat anti‐rabbit IgG‐HRP secondary antibody (Abcam, Cat #: sc‐2204 Lot #: B2216). All antibodies were used at the same concentration as the first blot. Membrane was visualized on the Azure imaging system.

### Single‐particle interferometric reflectance imaging

Both CVL‐derived and dendritic cell LK23‐derived EVs were diluted 1 : 1000 and incubated on ExoView (NanoView Biosciences, Brighton, MA, USA) chips that were printed with anti‐CD63 (BD Biosciences, Cat#: 556019) and anti‐CD81 (BD Biosciences, Cat #: 555675) antibodies. After incubation for 16 h, chips were washed as per the manufacturer's protocol and imaged in the ExoView scanner by interferometric reflectance imaging.

### Electron microscopy

Gold grids were floated on 2% paraformaldehyde‐fixed CVL‐derived samples for 2 min and then negatively stained with uranyl acetate for 22 s. Grids were observed with a Hitachi 7600 transmission electron microscope in the Johns Hopkins Institute for Basic Biomedical Sciences Microscope Facility.

### Total RNA isolation and quality control

RNA isolation workflow is shown in Fig. S1C. RNA lysis buffer was added into each sample as described above prior to freezing (−80 °C). Total RNA was isolated from thawed samples using the miRCURY RNA Isolation Kit‐Biofluids (Exiqon, Cat #: 300112. Lot #: 593‐84‐9n) as per the manufacturer's protocol with minor modifications as previously described [50]. Total RNA was eluted with 50 µL RNase‐free water and stored at −80 °C. As quality control, expression levels of several small RNAs (sRNAs) including snRNA U6, miR‐16‐5p, miR‐223‐3p, and the spiked‐in synthetic cel‐miR‐39 were assessed by TaqMan miRNA assays (Applied Biosystems/Life Technologies, Carlsbad, CA, USA) [51].

### miRNA profiling by TaqMan low‐density array

A custom 48‐feature TaqMan low‐density array (TLDA) was ordered from Thermo Fisher, with features chosen based on results of a human CVL pilot study (GVH and KWW, unpublished data). Stem‐loop primer reverse transcription and preamplification steps were conducted using the manufacturer's reagents as previously described [52] but with 14 cycles of preamplification. Real‐time quantitative PCR was performed with a QuantStudio 12K instrument (Johns Hopkins University DNA Analysis Facility). Data were collected using sds software and *C*_q_ values extracted with expression suite v1.0.4 (Thermo Fisher Scientific). Raw *C*_q_ values were adjusted by a factor determined from the geometric mean of 10 relatively invariant miRNAs. Normalizing to the geometric mean of multiple carefully selected housekeeping miRNAs is a biologically relevant and recommended normalization method [53]. The selection process for these invariant miRNAs was to (a) rank miRNAs by coefficient of variation; (b) remove miRNAs with high average *C*_q_ (> 30), non‐miRNAs, and those with low amplification score; (c) select the lowest‐CV member of miRNA families (e.g., the 17/92 clusters); and (d) pick the top 10 remaining candidates by CV: let‐7b‐5p, ‐miR‐21‐5p, ‐27a‐3p, ‐28‐3p, ‐29a‐3p, ‐30b‐5p, ‐92a‐3p, ‐197‐3p, ‐200c‐3p, and ‐320a‐3p.

### Individual RT‐qPCR assays

Individual TaqMan miRNA qPCR assays were performed as previously described [52] on all UC pellet samples from all animals across all weeks for miRs‐19a‐3p (Thermo Fisher Assay ID #000395), ‐186‐5p (Thermo Fisher Assay ID #002285), ‐451a‐5p (Thermo Fisher Assay ID #001105), ‐200c‐3p (Thermo Fisher Assay ID #002300), ‐222‐3p (Thermo Fisher Assay ID #002276), ‐193b‐3p (Thermo Fisher Assay ID #002367), ‐181a‐5p (Thermo Fisher Assay ID #000480), ‐223a‐3p (Thermo Fisher Assay ID #002295), ‐16‐5p (Thermo Fisher Assay ID #000391), ‐106a‐5p (Thermo Fisher Assay ID #002169), and ‐125b‐5p (Thermo Fisher Assay ID #00449). We also measured miR‐375‐3p (Thermo Fisher Assay ID #00564), which was not included on the array. Data were adjusted to Cqs of miR‐16‐5p.

### Blood cell isolation and monocyte‐derived macrophage culture

Total peripheral blood mononuclear cells (PBMCs) were obtained from freshly drawn blood from human donors under a Johns Hopkins University School of Medicine IRB‐approved protocol (JHU IRB #CR00011400). Blood was mixed with 10% ACD (Sigma‐Aldrich, Cat #: C3821 Lot #: SLBQ6570V) with gentle mixing by inversion. Within 15 min of draw, blood was diluted with equal volume of PBS + 2% FBS and gently layered onto room temperature Ficoll (Biosciences AB, Uppsala, Sweden, Cat #:17‐1440‐03 Lot #: 10253776) in Sepmate‐50 tubes (STEMCELL Technologies, Vancouver, BC, Canada, Cat #: 15450 Lot #: 06102016) and centrifuged for 10 min at 1200 ***g***. Plasma and PBMC fractions were removed, washed in PBS + 2% FBS, and pelleted at 300 ***g*** for 8 min. Pellets from five tubes were combined by resuspension in 10 mL red blood cell (RBC) lysis buffer (4.15 g NH_4_Cl, 0.5 g KHCO_3_, 0.15 g EDTA in 450 mL H_2_O; pH adjusted to 7.2–7.3; volume adjusted to 500 mL and filter‐sterilized); total volume was brought to 40 mL with RBC lysis buffer. After incubation at 37 °C for 5 min, the suspension was centrifuged at 400 ***g*** for 6 min at room temperature. The cell pellet was resuspended in macrophage differentiation medium with macrophage colony‐stimulating factor (M‐CSF) and 20% FBS (MDM20) to a final concentration of 2 × 10^6^ cells·mL^−1^. PBMCs were plated at 4 × 10^6^ cells per well in 12‐well plates and cultured in MDM20 for 7 days. One half of the total volume of medium was replaced on day 3. On day 7, cells were washed three times with PBS to remove nonadherent cells. The medium was replaced with macrophage differentiation medium with M‐CSF and 10% serum (MDM10) and cultured overnight prior to transfection.

### miRNA mimic transfection

Differentiated macrophages were transfected with 50 nm miRNA‐186‐5p (Qiagen, Foster City, CA, USA. Cat #: MSY0000456 Lot #: 286688176) using Lipofectamine 2000 (Invitrogen/Life Technologies, Carlsbad, CA, USA, Cat #: 11668‐019 Lot #:1467572) diluted in OptiMEM Reduced Serum Medium (Gibco, Grand Island, NY, USA, Cat #: 31985‐070 Lot #: 1762285). Controls included mock transfections and transfection of 50 nm double‐stranded siRNA oligo labeled with Alexa Fluor 555 (Invitrogen, Fredrick, MD, USA, Cat #: 14750‐100 Lot #: 1863892). Plates were incubated for 6 h at 37 °C. After incubation, successful transfection was confirmed by examining uptake of labeled siRNA with an Eclipse TE200‐inverted microscope (Nikon Instruments, Melville, NY, USA). Transfection medium was removed. The plates were washed with PBS and refed with 2 mL fresh MDM10 medium.

### HIV infection

HIV‐1 BaL stocks were generated from infected PM1 T lymphocytic cells and stored at −80 °C. 24 h after mimic or mock transfections; macrophages were infected with HIV BaL and incubated overnight (stock, 80 μg p24·mL^−1^, diluted to 200 ng p24·mL^−1^). At days 3, 6, and nine postinfection, 500 μL supernatant was collected for p24 release assays and cells were lysed with 600 μL mirVana lysis buffer for subsequent RNA isolation and analysis.

### HIV p24 antigen ELISA

Supernatant samples were lysed with Triton X (Perkin Elmer, Waltham, MA, USA, Cat #: NEK050B001KT Lot #: 990‐17041) at a final concentration of 1%. The DuPont HIV‐1 p24 Core Profile ELISA kit (Perkin Elmer, Cat #: NEK050B001KT Lot #: 990‐17041) was used as per the manufacturer's instructions to measure p24 concentration based on the provided standard.

### Total RNA isolation

Total RNA was isolated using the mirVana miRNA Isolation Kit as per the manufacturer's protocol (Ambion, Vilnius, Lithuania. Cat #: AM1560 Lot #: 1211082). Note that this procedure yields total RNA, not just sRNAs. After elution with 100 μL RNase‐free water, nucleic acid concentration was measured using a NanoDrop 1000 spectrophotometer (Thermo Fisher Scientific, Wilmington, DE, USA). RNA isolates were stored at −80 °C.

### HIV Gag RNA RT‐qPCR

Real‐time one‐step reverse transcription–quantitative PCR was performed with the QuantiTect Virus Kit (Qiagen, Cat #:211011 Lot #: 154030803). Each 25 μL reaction mixture contained 15 μL of master mix containing HIV‐1 RNA standard, 100 μm of FAM dye, and IBFQ quencher labeled Gag probe (5′ ATT ATC AGA AGG AGC CAC CCC ACA AGA 3′), 600 nm each of Gag1 forward primer (5′ TCA GCC CAG AAG TAA TAC CCA TGT 3′) and Gag2 reverse primer (5′ CAC TGT GTT TAG CAT GGT GTT T 3′), nuclease‐free water, and QuantiTect Virus RT mix, and 10 μL serial‐diluted standard or template RNA. No‐template control and no reverse transcriptase controls were included. Linear standard curve was generated by plotting the log copy number versus the quantification cycle (*C*_q_) value. Log‐transformed Gag copy number was calculated based on the standard curve.

### Data analysis

Data processing and analysis were conducted using tools from Microsoft Excel (geometric mean normalization), Apple Numbers, GraphPad Prism, the MultiExperiment Viewer, and r/BioConductor packages including pheatmap (http://CRAN.R‐project.org/package=pheatmap; quantile normalization, Euclidean distance, self‐organizing maps, self‐organizing tree algorithms, *k*‐means clustering).

## Results

### Abnormal menstrual cycle of SIV‐infected macaques and ovulation‐associated changes in CVL EV‐enriched particles

**Fig. 1:**
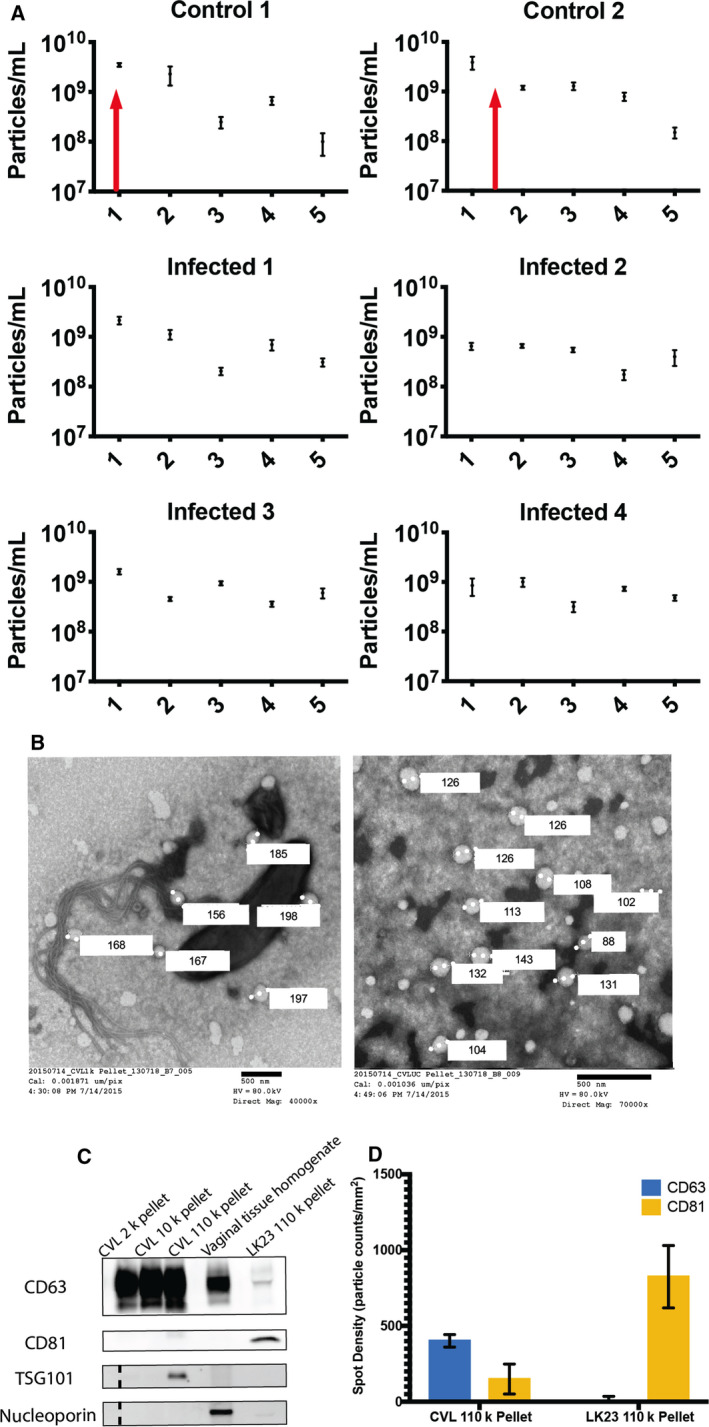
EV composition during the menstrual cycle. (A) Nanoparticle concentrations of CVL UC pellets monitored weekly over 5 weeks for two SIV‐negative (“control”) and four SIV‐infected rhesus macaques (Mean ± SD). Red arrows indicate time of ovulation for two control animals, which were absent for SIV‐infected animals. (B) Transmission electron micrographs of CVL pellets from the 10 000 ***g*** pellet (left) and 110 000 ***g*** pellet (right) confirm presence of bacteria and EVs/EV‐like particles, with several respective diameters indicated. Scale bar = 500 nm. (C) Western blot analysis suggests enrichment of EV markers CD63, CD81, and TSG101 in 110k pellet fraction of CVL from uninfected animals (*n* = 2). Vaginal tissue homogenate and DC (LK23) 110k pellet controls were also positive for CD63 and CD81. Nuclear marker nucleoporin was detected in tissue homogenate but not in putative EV samples. Dashed lines were added to indicate the grouping of the ladder and the samples on the TSG101 and nucleoporin blots. (D) SP‐IRIS confirmation of EV markers on CVL and DC EVs. Shown are averages of tetraspanin‐positive particles bound to anti‐CD63 and anti‐CD81 antibodies and detected by label‐free imaging (mean ± SD).

Plasma and CVL were collected from two control and four SIV‐infected macaques over the course of 5 weeks (Fig. S1). Amenorrhea (absence of menstruation) was observed for infected subjects (K. Mulka, *et al*, unpublished data). By NTA, CVL EV concentration in control animals increased during ovulation (Fig. 1A). Transmission electron microscopy was performed for representative fractions of CVL, revealing bacteria and large particles in the 10 000 ***g*** pellet (Fig. 1B). The 100 000 ***g*** pellet included apparent EVs up to 200 nm in diameter (Fig. 1C). EV markers (shown: CD63, CD81, and TSG101) were confirmed by Western blot (Fig. 1D). The nuclear marker nucleoporin was detected only in tissue samples (Fig. 1D). The relative EV tetraspanin profiles of both CVL and control EV samples were corroborated with single‐particle interferometric reflectance imaging: CVL EVs had a higher CD63 expression, and dendritic cell EVs had higher CD81 expression.

### TLDA reveals an extracellular miRNA profile of the cervicovaginal compartment

Based upon preliminary findings from a study of human CVL (Hancock and Witwer, unpublished data), we designed a custom TLDA to measure 47 miRNAs expected to be present in CVL, along with the snRNA U6. CVL from all subjects and at all time points was fractionated by stepped centrifugation to yield a 10 000 ***g*** pellet (10 K pellet), a 100 000 ***g*** pellet (UC pellet), and 100 000 ***g*** supernatant (UC supernatant). Total RNA from all fractions was profiled by TLDA. Raw (Fig. S2A), quantile normalized (Fig. S2B), and geometric mean‐adjusted *C*_q_ values (Fig. S2C) were subjected to unsupervised hierarchical clustering. This clustering did not reveal broad miRNA profile differences associated with sample collection time, menstruation, or SIV infection.

### Distribution of miRNAs across CVL fractions

**Fig. 2:**
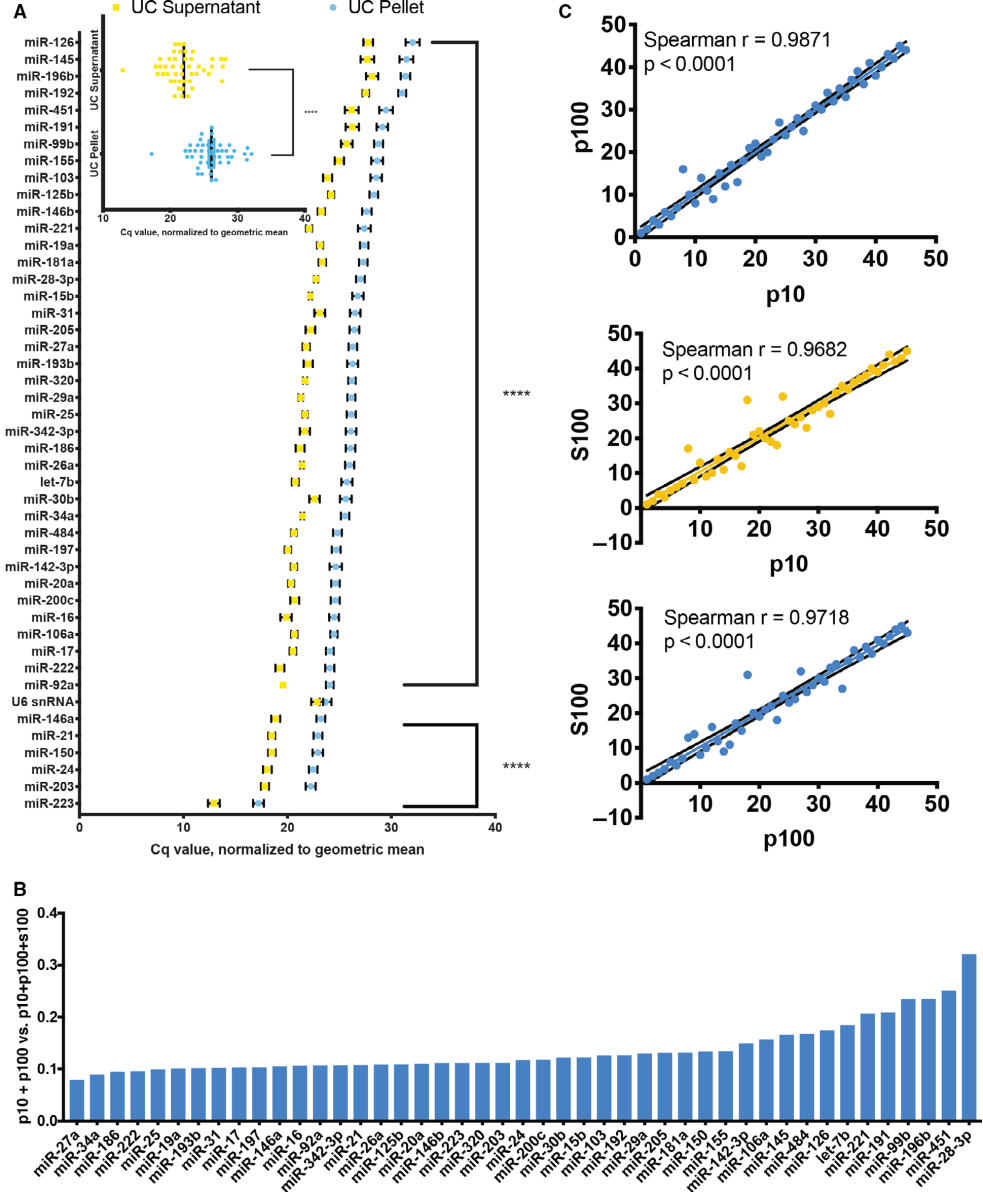
Relative abundance of miRNAs in different CVL fractions from all subjects (*n* = 6). (A) Abundant miRNAs in descending order based on *C*_q_ values normalized to the geometric mean for each sample. Inset: average of all miRNAs in UC pellet and UC supernatant. Error bars: SEM. (B) miRNA expression in EV‐enriched fractions (p10, p100) as a percentage of total estimated expression (p10 + p100+S100 by *C*_q_) in ascending order, from miR‐27a‐3p (7.9%) to miR‐28‐3p (32.0%). (C) miRNAs in each fraction (10 000 ***g*** pellet = p10, 110 000 ***g*** pellet = p100, 110 000 ***g*** supernatant = S100, and) are significantly correlated (*P* < 0.0001, Spearman).

Across the three examined CVL fractions (p10, p100, S100), the 10 most abundant miRNAs (lowest *C*_q_ values) were miRs‐223‐3p, ‐203a‐3p, ‐24‐3p, ‐150‐5p, ‐21‐5p, ‐146a‐5p, ‐92a‐3p, ‐222‐3p, ‐17‐5p, and ‐106a‐5p. The average normalized *C*_q_ value for each miRNA was greater (i.e., lower abundance) in the p100 than the s100 fraction (Fig. 2A and inset) and indeed in p10 and p100 combined (Fig. 2B), suggesting that most miRNA in CVL, as reported for various other body fluids, is found outside the EV‐enriched fractions. Considering all fractions, the differences between the EV‐enriched and EV‐depleted fractions were significant even after Bonferroni correction for all features except U6. On average, the s100 fraction contained 86.5% of the total miRNA from these three fractions. In the p10 fraction, the average miRNA was detected at 10.5% its level in the s100 fraction (SD = 5.7%). miR‐34a‐5p had the lowest (5.9%) and miR‐28‐3p the highest (33.7%) abundance compared with s100. In the p100 fraction, miRNAs were on average 5.6% (SD = 2.4%) as abundant as in s100. The least represented in p100 was miR‐27a‐3p (2.3%), and the best represented was again miR‐28‐3p (13.4%). Together, the content of the EV‐enriched fractions (p10 and p100) as a percentage of the total is shown in Fig. 2B for individual miRNAs. miRNA rank was significantly correlated across fractions, despite minor differences in order (Fig. 2C).

### qPCR Validation

**Fig. 3:**
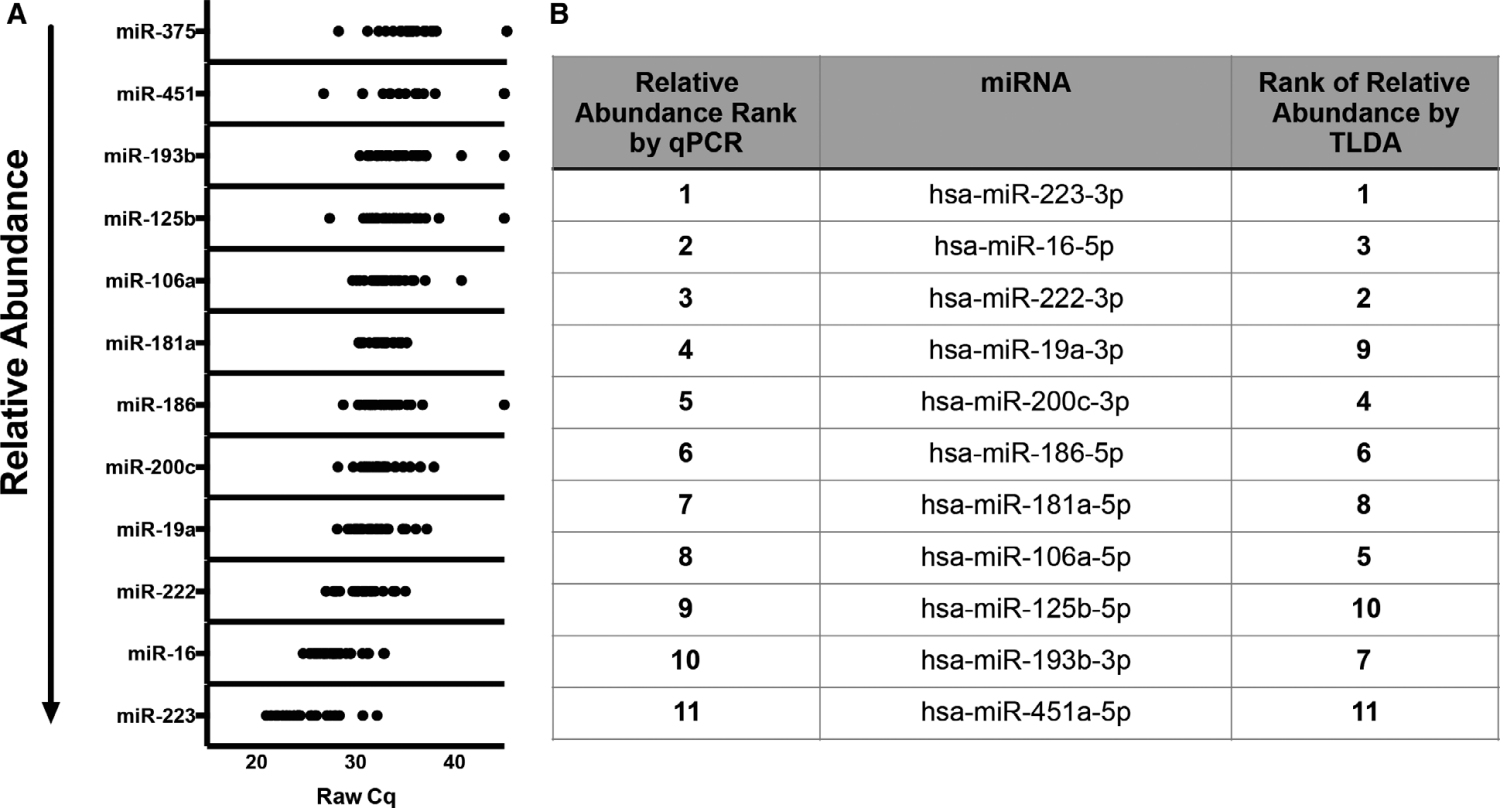
miRNA qPCR validation. (A) qPCR validation for UC pellet samples, all subjects (*n* = 6), and time points (individual dots). (B) Ranks of abundant miRNAs by qPCR and TLDA.

Individual stem‐loop RT/hydrolysis probe qPCR assays were used to verify TLDA results for eleven selected miRNAs plus miR‐375‐3p (not included on the array), which was also measured because of a reported association with goblet cells [54]. Some miRNAs were chosen due to high expression levels. miR‐181a‐5p was measured due to its association with endometrial cells [55, 56]. miR‐125b‐5p has been reported as a diagnostic marker of endometriosis [57]. Other miRNAs (miRs‐186‐5p, ‐451a‐5p, ‐200c‐3p, ‐222‐3p, ‐193b‐3p) were selected based on our previous experience and results from other studies evaluating miRNAs in the context of HIV‐1 and SIV infections. Results of qPCR assays, adjusted by miR‐16‐5p for each sample (since we found relatively low qPCR variation of miR‐16‐5p, a commonly used normalizer [58]), are shown in Fig. 3A. Figure 3B compares miRNA ranks (1–11) by TLDA and individual qPCR, which are generally in concordance. Note that expression of RBC miRNA miR‐451a‐5p was low, suggesting minimal contamination from blood for most samples.

### miRNA association with retroviral infection status

**Fig. 4:**
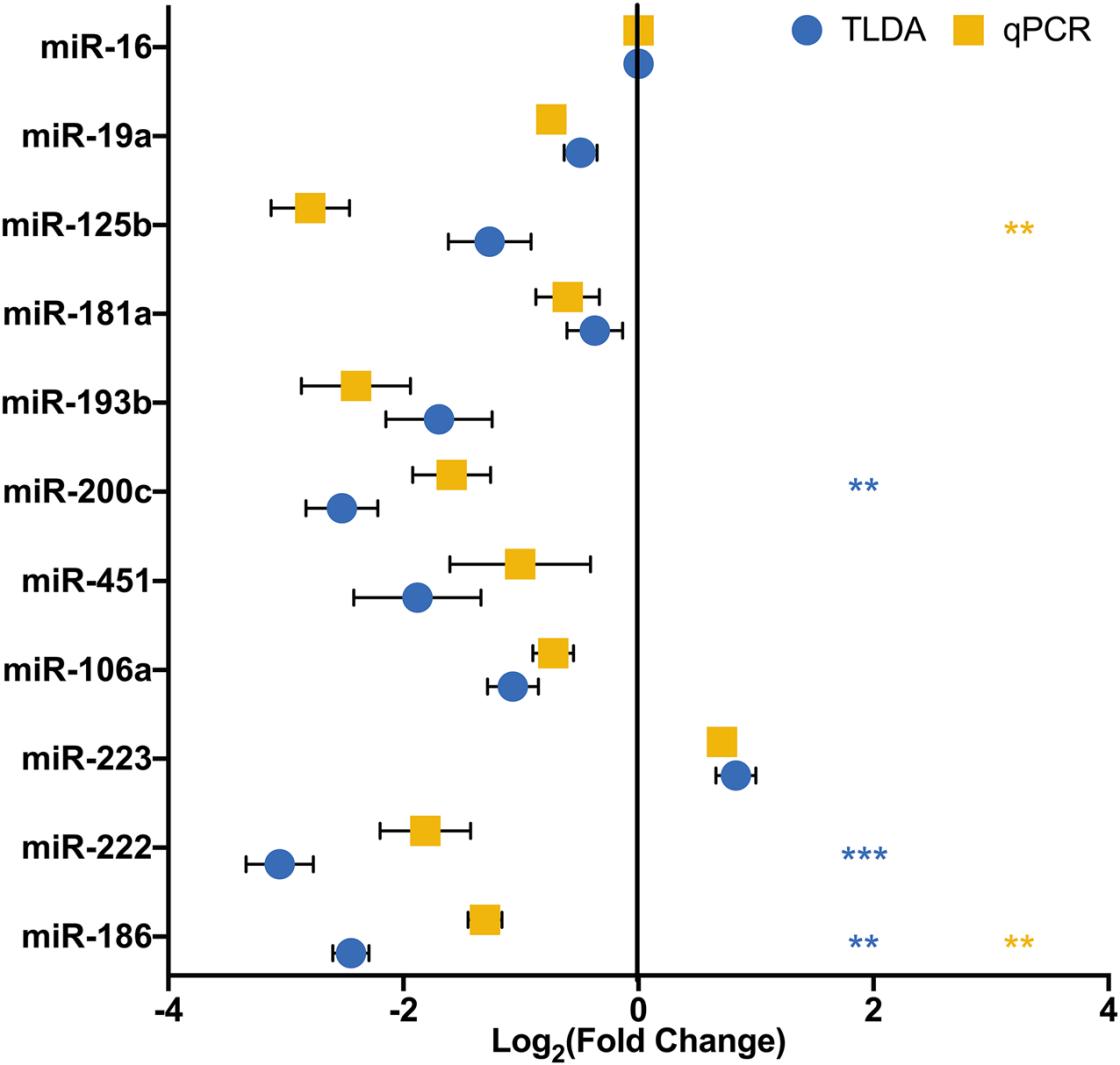
miR‐186‐5p downregulation: SIV. miR‐186‐5p fold change for all infected animals (*n* = 4) was determined using ∆∆*C*_t_ method using miR‐16 and uninfected animals (*n* = 2) as controls. Log_2_ (fold change) for both TLDA and qPCR analyses was plotted for 11 selected validation miRNAs. Statistical analyses were performed on ∆*C*_t_ values. For TLDA, miRs‐186, ‐222, and ‐200c were significantly less abundant in the CVL p100 fraction of infected subjects (mean ± SEM, multiple *t*‐test, Bonferroni–Dunn correction), **P < 0.01, ***P < 0.001. For qPCR analysis, miRs‐186 and ‐125b were significantly less abundant (multiple *t*‐test, Bonferroni–Dunn correction), **P < 0.01, ***P < 0.001.

An association of miRNA abundance with infection status could yield novel biomarkers as well as clues to roles of miRNA in modulating infection. However, the small number of subjects in our study was a challenge. Nevertheless, by considering all subjects and time points together for both infected and uninfected subjects, microarray data suggested a slightly reduced abundance of miRs‐186‐5p, ‐222‐3p, and ‐200c‐3p in infected samples (Fig. 4A) based on statistical analysis of ∆*C*_q_ values, while qPCR revealed differential abundance of miRs‐186‐5p and ‐125b‐5p (Fig. 4B). miR‐186‐5p was thus identified by both techniques as potentially associated with retroviral infection.

### miR‐186‐5p transfection has minimal effects on cellular HIV RNA abundance but reduces p24 release from monocyte‐derived macrophages

**Fig. 5:**
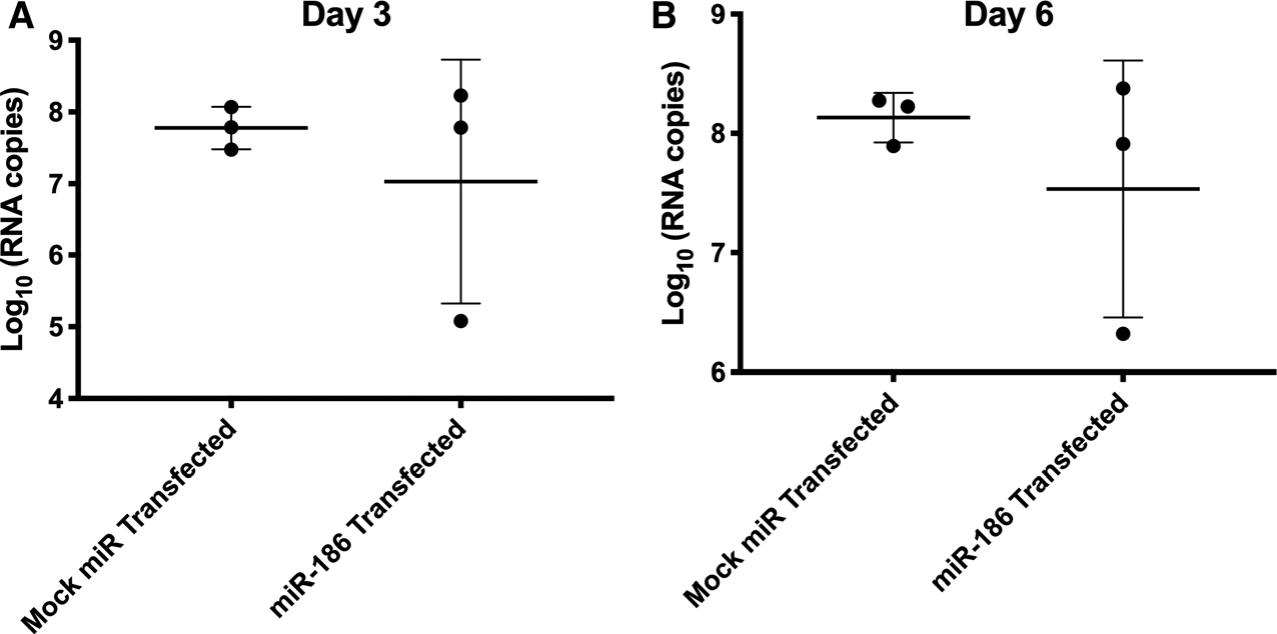
miRNA‐186‐5p mimic transfection inconsistently suppresses HIV‐1 gag mRNA production. Apparent downregulation of gag mRNA (qPCR assay with standard curve) was observed in miR‐186‐transfected monocyte‐derived macrophages from only one of three donors compared with mock miRNA‐transfected cells. Overall, results were insignificant by *t*‐test (mean ± SEM), *P* > 0.1, with multiple replicates of cells from human donors (*n* = 3).

To assess a possible influence of miR‐186‐5p (“miR‐186”) on retroviral replication, we introduced double‐stranded miR‐186‐5p mimic or control RNA into monocyte‐derived macrophages derived from three donors 24 h before infecting the cells or not with HIV. Upon M‐CSF stimulation, the primary macrophages should be activated toward the alternative M2 phenotype [59, 60, 61]. Post‐infection, EVs and miRNAs were mostly likely released from uninfected and infected macrophages alike. At days 3 and 6 postinfection, we quantitated full‐length HIV‐1 transcript using a gag qPCR with standard curve. In cells from only one of three donors were fewer HIV‐1 copies associated with miR‐186‐5p mimic transfection (Fig. 5). Overall, there was no statistically significant difference in HIV RNA between the conditions.

**Fig. 6:**
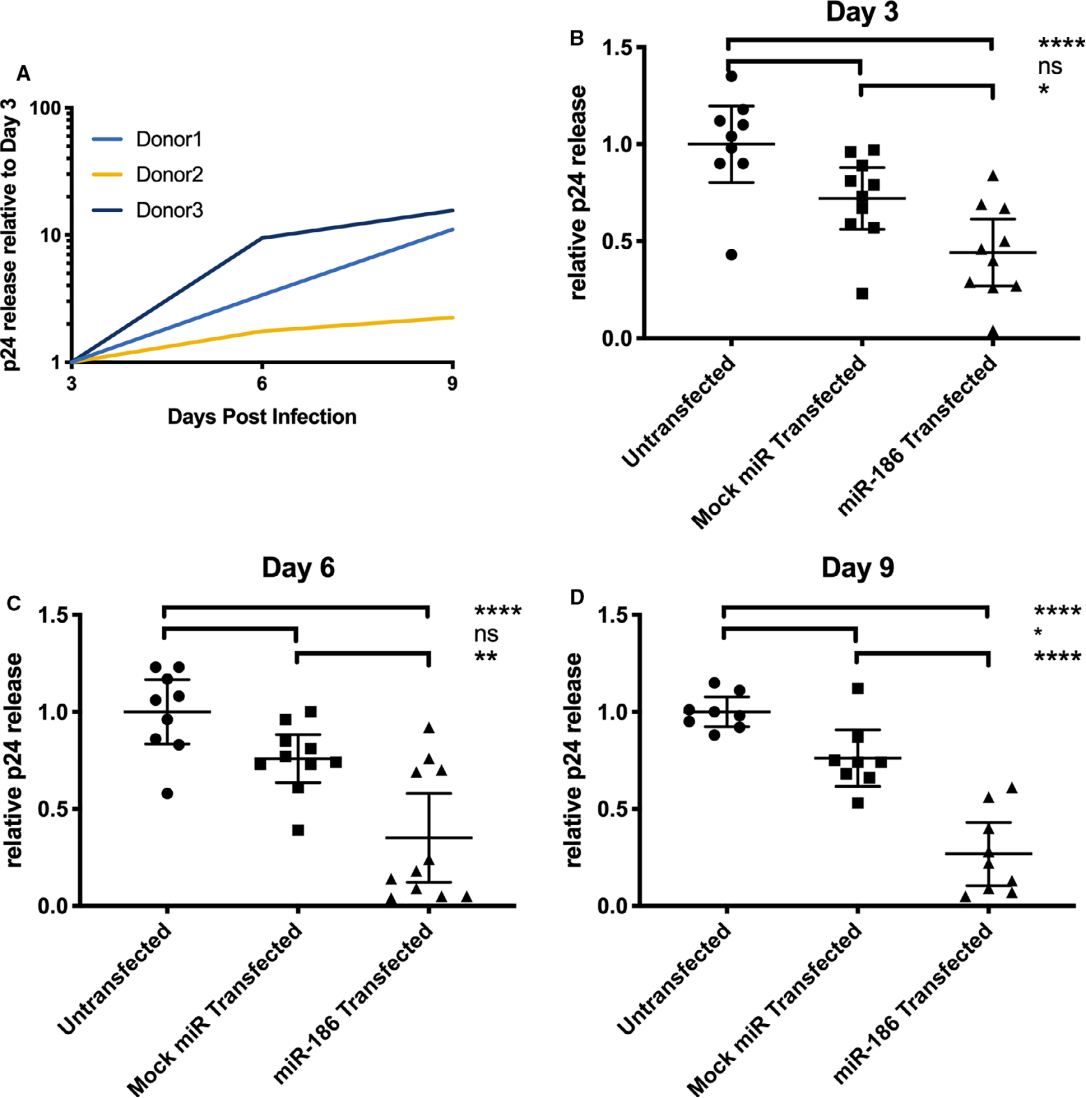
miRNA‐186‐5p inhibits p24 release. Monocyte‐derived macrophages from human donors were infected with HIV‐1 BaL. (A) p24 production increased > 2‐fold for all donors from 3 to 9 days postinfection (dpi), untreated cells. (B–D) Transfection of miR‐186‐5p mimic was associated with a decrease of p24 release compared with untransfected controls and mock miRNA mimic‐transfected controls at the indicated time points; ns = not significant, *P < 0.05, **P < 0.01, ****P < 0.0001 (mean ± SEM, ANOVA followed by Bonferroni correction for multiple tests). Results were from a total of eight to 11 replicate experiments with cells from all human donors (*n* = 3).

However, at the same time points and also out to 9 days postinfection, a different result was seen for capsid p24 release into the supernatant. For infected but untransfected cells, measurable p24 was observed by 3 dpi, and p24 counts increased by twofold or more by 9 dpi (Fig. 6A) for multiple replicate experiments with cells from three donors. Compared with infected, untreated controls, mock‐transfected cells (not shown), and cells transfected with a negative control RNA (labeled with a fluorophore to assess transfection efficiency), miR‐186‐5p transfection was associated with a significant decline of released p24 at all time points (ANOVA with Bonferroni correction; Fig. 6B–D). The negative control condition showed a suppressive trend that reached nominal significance at 9 dpi. However, miR‐186‐associated suppression was significantly greater at all time points.

### p24 inhibition by miR‐186‐5p is correlated with transfection efficiency

**Fig. 7:**
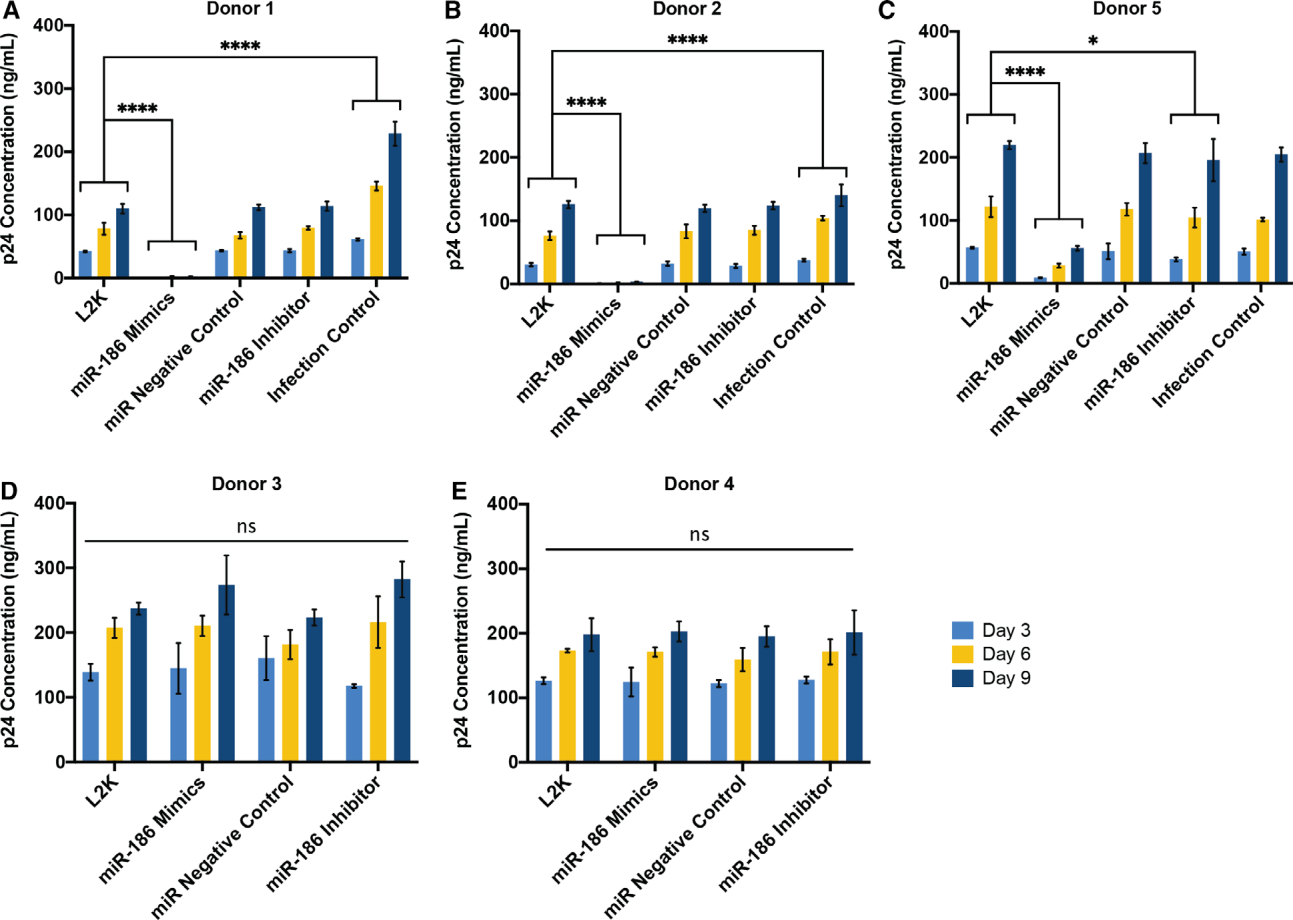
miRNA‐186‐5p inhibits p24 release in a donor‐specific manner. Monocyte‐derived macrophages from human donors were infected with HIV‐1 BaL. (A–C) Compared with mock‐transfected controls, transfection of miR‐186 mimic was associated with a significant decrease of p24 production from 3 to 9 days postinfection, respectively (dpi) in donors 1, 2, and 5 (biological replicate *n* = 3, technical replicate *n* = 2). (D, E) For donors 3 and 4, transfection of miR‐186 mimic was ineffective in inhibiting p24 release compared with mock‐transfected controls (biological replicate *n* = 3, technical replicate *n* = 2); ns = not significant, *P < 0.05, ****P < 0.0001 (mean ± SEM, two‐way ANOVA followed by Bonferroni correction for multiple tests).

**Fig. 8:**
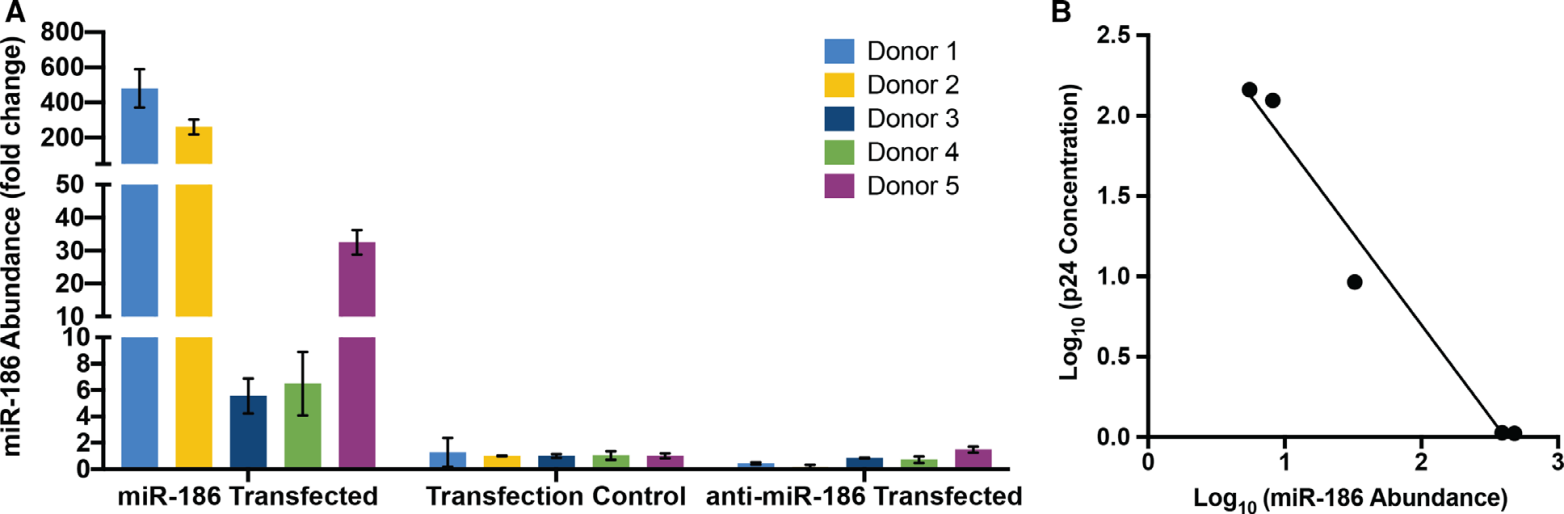
miR‐186‐5p abundance post‐transfection and correlation with p24 release. (A) Abundance of miR‐186‐5p in macrophages post‐transfection, as assessed by qPCR and compared (fold change) with the average of control macrophages (Mean ± SD). (B) Correlation of macrophage miR‐186‐5p and p24 concentration released in supernatant 3 days postinfection. *P*(two‐tailed) = 0.0019 (correlation), *R* ^2 ^= 0.9731 (linear regression).

Despite the statistical significance of miR‐186‐5p‐associated p24 inhibition, substantial variability was observed, including between donors/experiments; we therefore hypothesized that either donor‐ or experiment‐specific factors were responsible for the variability. The transfection experiments were repeated using macrophages from five additional donors (labeled 1–5). While significant but variable inhibition of p24 release after miR‐186‐5p transfection was observed for three donors (1, 2, and 5), little or no inhibition was seen for donors 3 and 4 (Fig. 7). It should be noted that miR‐186‐5p antisense inhibitors were also introduced in these experiments. While they did not significantly increase HIV p24 release (Fig. 7), they also did not achieve a consistent knockdown of native miR‐186‐5p (Fig. 8A).

One experimental variable that could affect the degree of inhibition is the efficiency with which the miRNA mimic is delivered into the cells. Since this variable was not assessed in our previous experiments, we measured it for the five new experiments. Despite using the same nominal concentrations of miRNA mimics for our experiments, a nearly 100‐fold range of miR‐186‐5p concentration was observed between the lowest and highest efficiency transfections (Fig. 8A), which increased miR‐186‐5p levels from around 5‐fold to nearly 500‐fold, respectively. Strikingly, the miR‐186‐5p level was inversely correlated with released p24 across these five donors (Fig. 8B).

## Discussion

Cervicovaginal lavage EVs and exRNPs, like EVs in the uterus [62, 63], may offer information about the health of the reproductive tract and may also facilitate or block transmission of infectious agents. Proteomic analyses of human [64] and rhesus macaque [65] CVL have suggested a core proteome and a highly variable proteome that responds to changes in pregnancy status, menstruation, infection, and other stressors. However, exRNA and EV profiles are less understood in this compartment. Thus, one major finding of this study is a partial profile of miRNAs of EV‐enriched and EV‐depleted fractions of CVL fluid of primates. We report that EVs can be liberated from vaginal secretions by lavage and that these EVs can be concentrated using a standard stepped centrifugation procedure, with enrichment of positive (membrane‐associated) markers while a cellular negative control was not detected.

Both EV‐replete and EV‐depleted fractions of CVL contained abundant miRNA. As reported for other biological fluids [37, 38], miRNA concentration was highest in the EV‐depleted CVL fractions, not in EV‐enriched UC pellets, consistent with packaging of most extracellular miRNA into exRNPs; the function, if any, of extracellular miRNAs in the cervicovaginal tract of healthy individuals remains to be determined. We observed minimal differences in extracellular miRNA profiles between SIV‐infected and uninfected subjects or, surprisingly, even during the menstrual cycle, suggesting a certain stability of extracellular miRNA in the compartment. Correlation of miRNA concentrations in EV‐depleted and EV‐replete fractions was also apparent. Based on relative abundance compared with miRNAs of other cellular/tissue origins (e.g., heart‐ and lung‐specific miR‐126, kidney‐specific miR‐196b, and liver‐specific miR‐192) [66, 67], miRNAs in EVs and exRNPs of CVL are likely derived from epithelial cells (including goblet cells) and cells of the immune system (as suggested, e.g., by myeloid‐enriched miR‐223 and lymphocyte‐enriched miR‐150) [68]. Of the most abundant miRNAs we identified, some have been ascribed tumor‐suppressive roles in cancers [69, 70, 71, 72, 73, 74, 75]. Also, miR‐223 and miR‐150 have been described as “anti‐HIV” miRNAs [76] among a variety of reported antiretroviral sRNAs, both host and viral [77, 78, 79, 80, 81, 82]. Given their relative abundance in the vaginal tract, a common site for HIV infection, these miRNAs may contribute to antiviral defenses.

Along these lines, a second major finding of this study is a possible role for miR‐186‐5p in antiretroviral defense, bolstered by the observation that exogenous miR‐186‐5p transfection efficiency correlates inversely with HIV p24 release. Previous publications have identified protein constituents in the CVL with anti‐HIV efficacies (e.g., [7, 21, 22]). Our identification of miRNA as a potential anti‐HIV agent adds an element of complexity to the picture of tissue‐specific antiretroviral defense. In contrast with an early report of direct binding of host miRNAs to retroviral transcripts and subsequent suppression [76], it now appears that this mechanism of suppression may be relatively uncommon [83]. Anti‐HIV miRNAs may be more likely to exert effects through control of host genes instead (e.g., [84]). Our data also support the conclusion that reduction of HIV RNA levels is not the main mechanism for miR‐186‐mediated suppression of HIV release.

How, then, might miR‐186‐5p, whether endogenous or exogenous (therapeutically introduced) contribute to antiretroviral effects? Combining several miRNA target prediction, validation, and enrichment analysis approaches [85, 86, 87, 88, 89, 90, 91], we noticed a few putative miR‐186‐5p targets and related pathways that may merit follow‐up. One target of miR‐186‐5p that was validated experimentally by multiple methods is FOXO1 [92], an important contributor to apoptosis but also immunoregulation via IFNγ pathways. Another prominent validated target, P2X7R [93], is involved in membrane budding, T‐cell‐mediated cytotoxicity, cellular response to extracellular stimuli, and T‐cell homeostasis/proliferation. There is also evidence that miR‐186‐5p targets the HIV coreceptor CXCR4 [94]. Pathway enrichment analyses [90, 91] suggest that miR‐186‐5p targets participate significantly in infection‐related networks, including prion diseases, viral carcinogenesis, and responses to measles and herpes simplex virus infections. Although miRNA target prediction algorithms are imperfect, and validation efforts are of varying quality [95, 96], these findings may shed some light on how miR‐186‐5p is involved in responses to HIV.

We would like to emphasize several aspects of the study that open the door to future research:

- We used stepped ultracentrifugation without density gradients because of the small sample volumes available. Although stepped ultracentrifugation remains a widely used method for EV enrichment [49, 97], subsequent gradients or alternative isolation methods could be attempted with larger volume samples to increase purity in future. Possibly, our study overestimates the abundance of miRNAs in CVL EVs, and differential packaging into EVs and exRNPs is masked by contamination of our EV preps with exRNPs.
- Our qPCR array approach and focus on miRNAs leaves room for additional work. While we are confident that our array captured most of the abundant miRNAs in CVL, sequencing short and longer RNAs could reveal additional markers.
- The small number of subjects and the absence of obvious menstrual cycle in infected subjects preclude strong conclusions about EV or miRNA associations with either infection or the menstrual cycle. For example, we did not observe the expected increase in miR‐451a or other RBC‐specific miRNAs during menstruation. However, since only two animals showed evidence of cycling, experiments with more subjects and larger sample volumes are needed.
- Our previous criticisms of miRNA functional studies [98] also apply to our results here. Additional work is needed to assess the potential of miR‐186‐5p to regulate retrovirus production at endogenous levels, for example by showing that it is present in active RNPs [99] and that it interacts directly with specific host or viral targets. However, it is also important to note that miR‐186‐5p could have therapeutic benefit even if it must be delivered at supraphysiologic concentrations. Finally, it is possible, but must be demonstrated, that miR‐186‐5p acts in a paracrine fashion via EV or exRNP shuttles.
- We have investigated the effects of miR‐186‐5p only in monocyte‐derived macrophages. We chose to begin with this cell type because of the abundance of miR‐223 and the known role of macrophages in the epithelium. We would like to reiterate the importance of other cell types in the vaginal tissue during HIV‐1 infection [100, 101]; thus, this antiviral effect of miR‐186‐5p should also be investigated in other cell types.

Overall, the results presented here support further development of CVL and its constituents as a window into the health of the cervicovaginal compartment in retroviral infection and beyond. Furthermore, delivery of miR‐186‐5p could act to suppress retrovirus release.

## Conflicts of interest

The authors have no competing interests to declare. The funding organization(s) played no role in the study design; in the collection, analysis, and interpretation of the data; in the writing of the report; or in the decision to submit the report for publication.

## Author contributions

ZZ, DCM, GVH, KAMP, and KWW involved in conceptualization; ZZ and KWW involved in data curation; ZZ, DCM, and KWW made formal analysis; KWW acquired funding; ZZ, DCM, KM, ZL, and BHP involved in the investigation; ZZ, KM, GVH, KAMP, and KWW contributed to methodology; KWW administrated the project; KAMP and KWW facilitated the resources; ZL and KWW supervised the study; ZZ, DCM, and KWW involved in visualization; KWW wrote the original draft; ZZ, DCM, and KWW Writing – review and editing.

## Supporting information


**Fig. S1.** Specimen collection and sample processing workflow.
**Fig. S2.** miRNA profile of CVL fractions.
**Fig. S3.** miRNA‐186‐5p suppresses HIV‐1 gag mRNA production on Day 6 and inhibits p24 release on Day 3–6.
**Table S1.** Recovered volumes: CVL.
**Table S2.** NTA dilution factors, CVL.

## Data Availability

Array data have been deposited with the Gene Expression Omnibus [102] as GSE107856. Data in other formats are available upon request. To the extent that sample quantities would allow, the minimal information for studies of extracellular vesicles recommendations for EV studies were followed [24], and the EV experiments have been registered with the EV‐TRACK knowledgebase [103] with preliminary EV‐TRACK code XL5296IL.
