# Supplementary material for: miRNA profiling of primate cervicovaginal lavage and extracellular vesicles reveals miR‐186‐5p as a potential antiretroviral factor in macrophages
Source: FEBS Open Bio. 2020 Sep 11;10(10):2021–39. doi: 10.1002/2211-5463.12952 (PMC7530394; doi:10.1002/2211-5463.12952)
Supplement: Supplementary file 1 — Fig. S1. Specimen collection and sample processing workflow. Fig. S2. miRNA profile of CVL fractions. Fig. S3. miRNA‐186‐5p suppresses HIV‐1 gag mRNA production on Day 6 and inhibits p24 release on Day 3–6. Table S1. Recovered volumes: CVL. Table S2. NTA dilution factors, CVL. [file FEB4-10-2021-s001.docx]

**Supporting Documents**


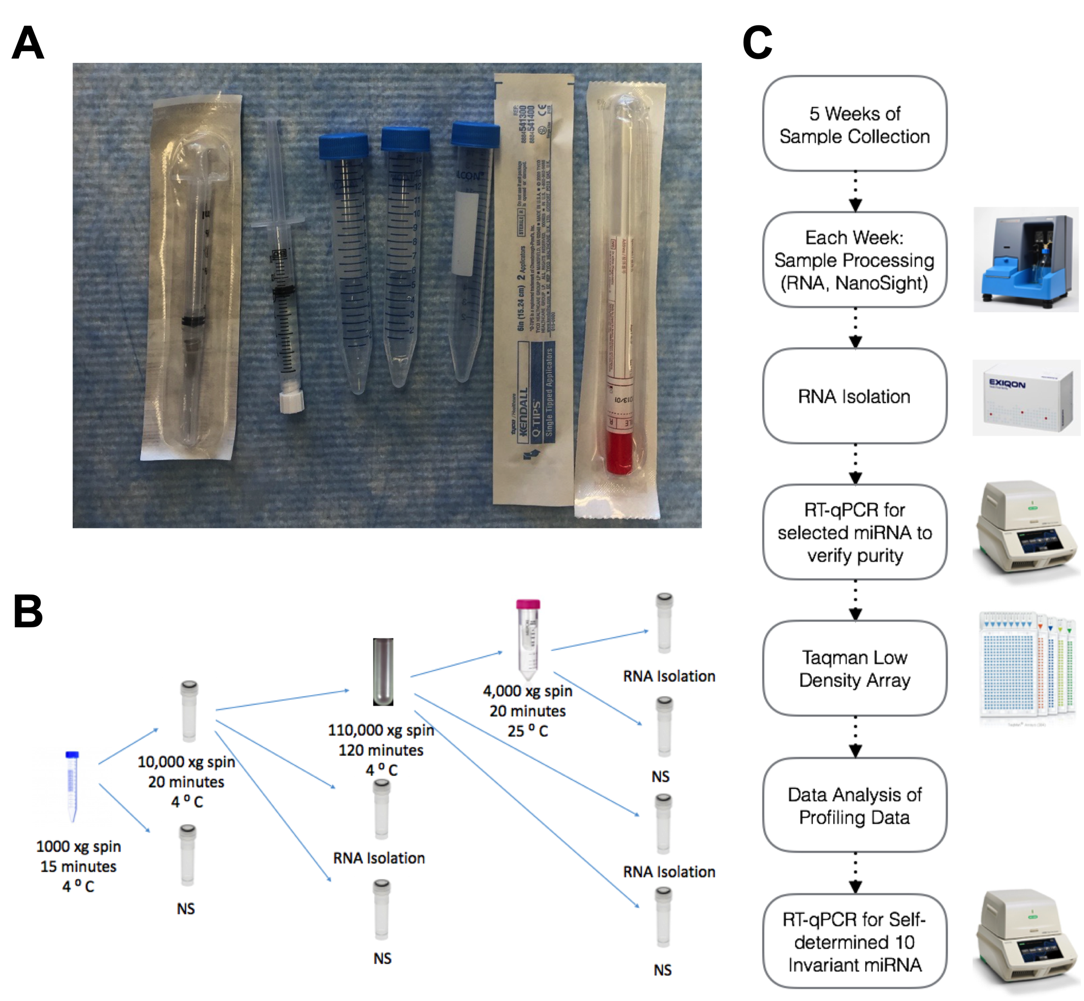


**Supplemental Figure 1.** Specimen Collection and Sample Processing Workflow. A) Materials used to collect blood, cervicovaginal lavage (CVL) and vaginal swab (VS—not discussed here) samples. CVL was collected with a syringe containing 3 mL of PBS. PBS was injected into the vaginal cavity and then withdrawn into the same syringe. VS samples were obtained serially using two cotton applicators to swab the vaginal canal. Each swab was placed in 2mL of 1× PBS and the swab material was removed from the cotton by swirling in PBS and then pressing to the side of the conical. B) Stepped ultracentrifugation to enrich for extracellular vesicles (EVs). C) Workflow including miRNA profiling by TaqMan low-density array. 10 miRNAs were verified by qPCR.


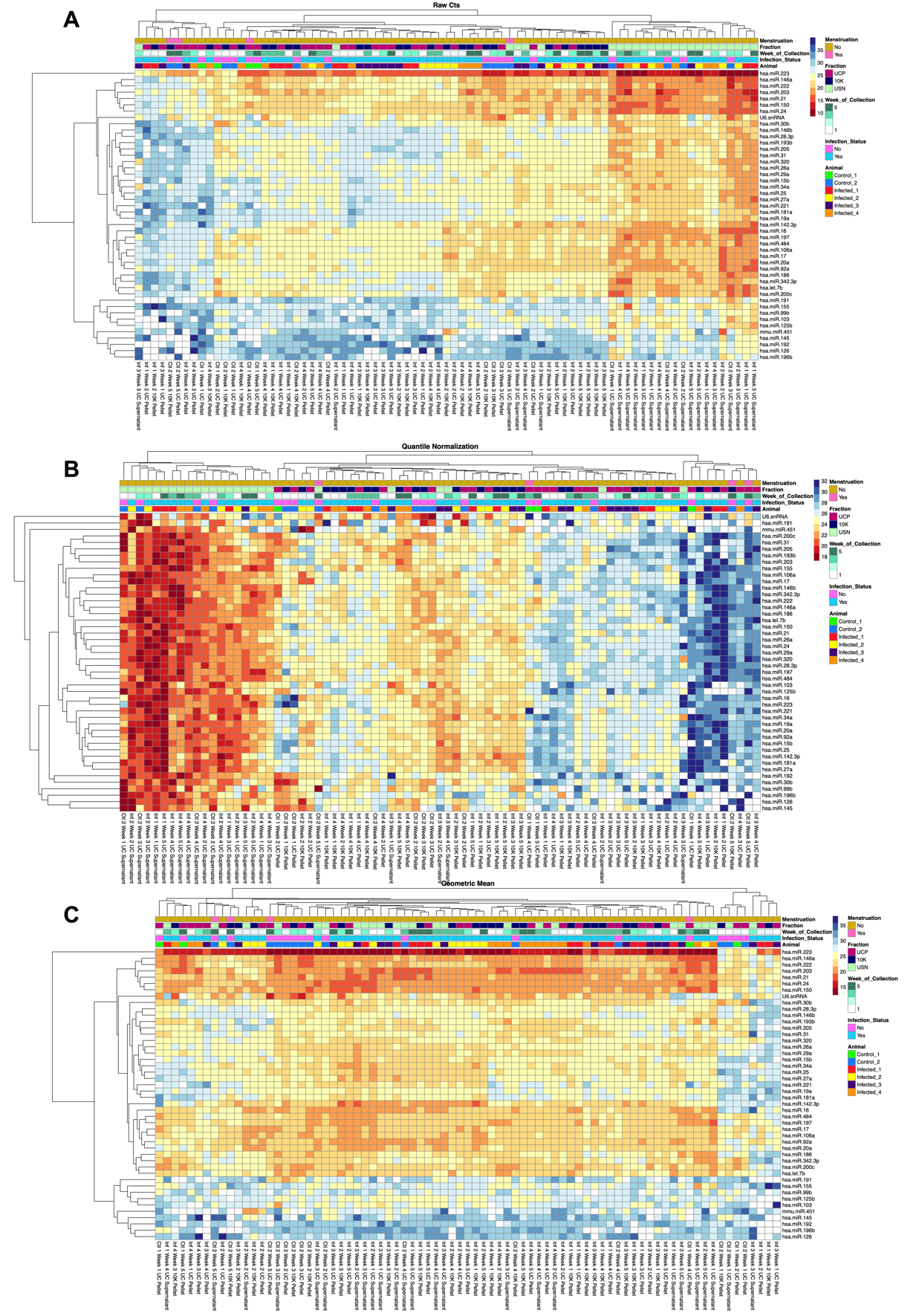


**Supplemental Figure 2**. Figure 2. miRNA profile of CVL fractions. miRNA profiles were determined by custom TaqMan low-density array (TLDA). Hierarchical clustering of samples and features (Pearson correlation, average linkage) of data: raw (A) or normalized by (B) quantiles or (C) a geometric mean approach as described in the methods. Abundance scale: red (high) to low (blue).


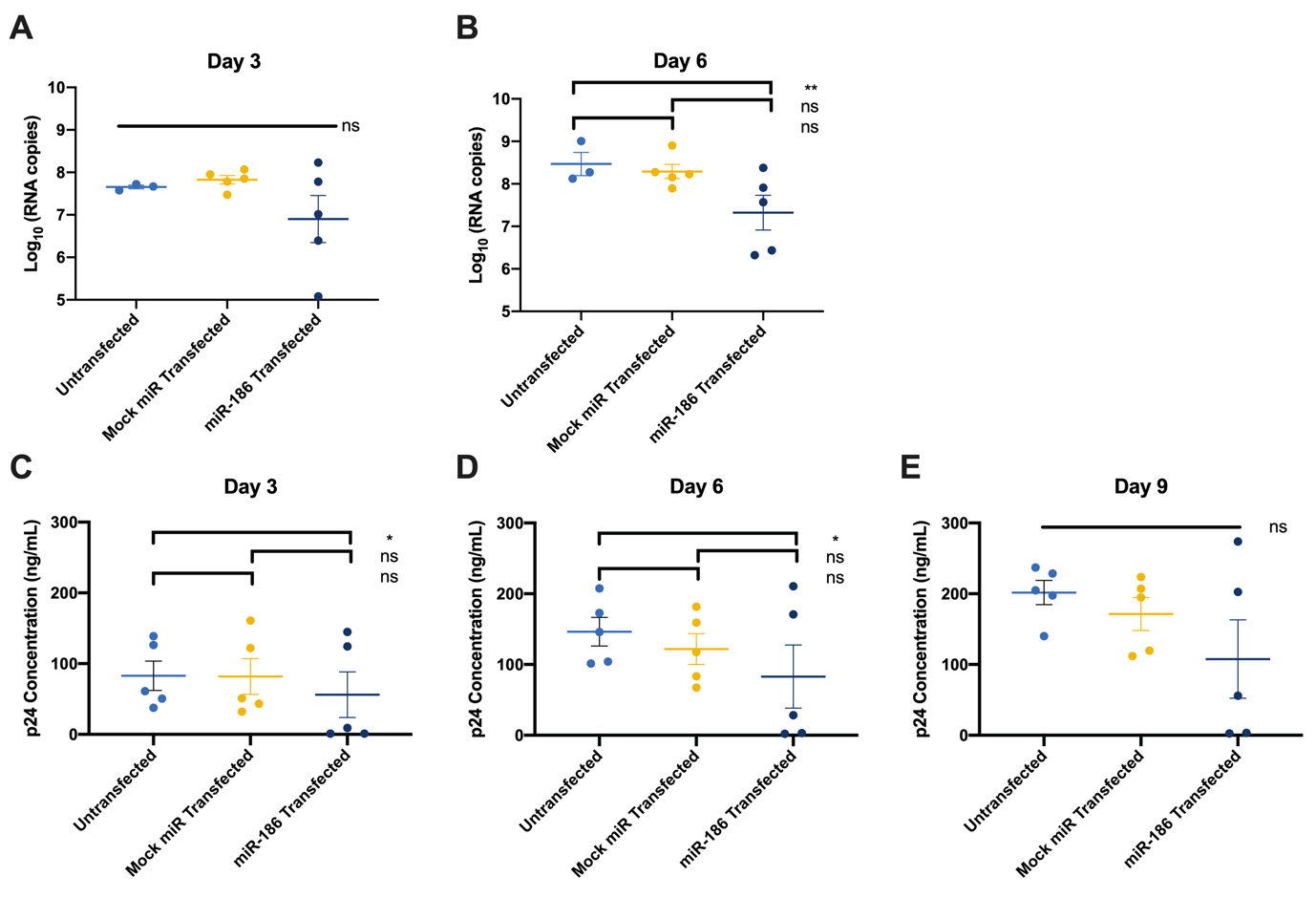


**Supplemental Figure 3.** miRNA-186-5p suppresses HIV-1 gag mRNA production on Day 6 and inhibits p24 release on Day 3-6. A-B) Apparent downregulation of gag mRNA (qPCR assay with standard curve) was observed on Day 6 between miR-186-transfected monocyte-derived macrophages and untransfected cells by t-test; ns=not significant, * p<0.05, ** p<0.01 (Mean ± SEM), with multiple replicates of cells from 5 human donors. C-E) Transfection of miR-186-5p mimic was associated with a decrease of p24 release compared with untransfected controls on Day 3 and 6; ns=not significant, * p<0.05, ** p<0.01, (Mean ± SEM, ANOVA followed by Bonferroni correction for multiple tests). Results were from a total of 5 human donors.

**Supplemental Table 1. Recovered volumes: CVL**

| **Subject** | **Week 1** | **Week 2** | **Week 3** | **Week 4** | **Week 5** | |  |
| --- | --- | --- | --- | --- | --- | --- | --- |
| **Control 1** | 1 mL | 2 mL | 1 mL | 1 mL | 1 mL |  |  |
| **Control 2** | 3 mL | 0.7 mL | 2 mL | 0.5 mL | 1.25 mL |  |  |
| **Infected 1** | 1 mL | 0.2 mL | 0.5 mL | 0.3 mL | 1.5 mL |  |  |
| **Infected 2** | 1.5 mL | 1.5 mL | 2 mL | 0.8 mL | 1.75 mL |  |  |
| **Infected 3** | 0.5 mL | 0.6 mL | 2.8 mL | 0.8 mL | 2 mL |  |  |
| **Infected 4** | 0.8 mL | 0.3 mL | 0.6 mL | 0.3 mL | 1.5 mL |  |  |

**Supplemental Table 2. NTA dilution factors, CVL**

|  | **Time point** | **Subject** | | | | | |
| --- | --- | --- | --- | --- | --- | --- | --- |
|  |  | **Control 1** | **Control 2** | **Infected 1** | **Infected 2** | **Infected 3** | **Infected 4** |
| CVL (UC Supernatant) | Week 1 | 1:25 | 1:50 | 1:25 | 1:25 | 1:25 | 1:25 |
|  | Week 2 | 1:100 | 1:5 | 1:25 | 1:10 | 1:5 | 1:5 |
|  | Week 3 | 1:10 | 1:5 | 1:5 | 1:5 | 1:10 | 1:10 |
|  | Week 4 | 1:10 | 1:5 | 1:10 | 1:5 | 1:10 | 1:10 |
|  | Week 5 | 1:5 | 1:10 | 1:10 | 1:10 | 1:5 | 1:5 |
| CVL (UC Pellet) | Week 1 | Neat | 1:5 | 1:5 | 1:5 | 1:5 | 1:5 |
|  | Week 2 | 1:10 | 1:10 | 1:5 | 1:5 | 1:5 | 1:5 |
|  | Week 3 | 1:5 | 1:5 | 1:5 | 1:5 | 1:5 | 1:5 |
|  | Week 4 | 1:5 | 1:5 | 1:5 | 1:5 | 1:5 | 1:5 |
|  | Week 5 | 1:5 | 1:5 | 1:5 | 1:5 | 1:5 | 1:5 |
| Abbreviations: CVL = cervicovaginal lavage; UC = ultracentrifugation | | | | | | | |
